# Macrolides decrease the minimal inhibitory concentration of anti-pseudomonal agents against *Pseudomonas aeruginosa* from cystic fibrosis patients in biofilm

**DOI:** 10.1186/1471-2180-12-196

**Published:** 2012-09-08

**Authors:** Larissa Lutz, Dariane Castro Pereira, Rodrigo Minuto Paiva, Alexandre Prehn Zavascki, Afonso Luis Barth

**Affiliations:** 1 Unidade de Microbiologia, Serviço de Patologia Clínica, Hospital de Clínicas de Porto Alegre, Porto Alegre, Brazil; 2 Unidade de Biologia Molecular, Serviço de Patologia Clínica, Hospital de Clínicas de Porto Alegre, Porto Alegre, Brazil; 3 Infectious-Diseases Service, Hospital de Clínicas de Porto Alegre, Porto Alegre, Brazil; 4 Serviço de Patologia Clínica, Hospital de Clínicas de Porto Alegre, Porto Alegre, Brazil

**Keywords:** Antimicrobial susceptibility test, Biofilm, Azithromycin, Clarithromycin

## Abstract

**Background:**

Biofilm production is an important mechanism for bacterial survival and its association with antimicrobial resistance represents a challenge for the patient treatment. In this study we evaluated the *in vitro* action of macrolides in combination with anti-pseudomonal agents on biofilm-grown *Pseudomonas aeruginosa* recovered from cystic fibrosis (CF) patients*.*

**Results:**

A total of 64 isolates were analysed. The biofilm inhibitory concentration (BIC) results were consistently higher than those obtained by the conventional method, minimal inhibitory concentration, (MIC) for most anti-pseudomonal agents tested (ceftazidime: *P* = 0.001, tobramycin*: P* = 0.001, imipenem: *P* < 0.001, meropenem: *P* = 0.005). When macrolides were associated with the anti-pseudomonal agents, the BIC values were reduced significantly for ceftazidime (*P <* 0.001) and tobramycin (*P <* 0.001), regardless the concentration of macrolides. Strong inhibitory quotient was observed when azithromycin at 8 mg/L was associated with all anti-pseudomonal agents tested in biofilm conditions.

**Conclusions:**

*P. aeruginosa* from CF patients within biofilms are highly resistant to antibiotics but macrolides proved to augment the *in vitro* activity of anti-pseudomonal agents.

## Background

The main cause of morbidity and mortality in cystic fibrosis (CF) is chronic lung disease caused by a vicious cycle of infection and inflammation which leads to progressive deterioration of pulmonary function, respiratory failure, and death [1]. *Pseudomonas aeruginosa* is the main bacteria associated with pulmonary disease in CF*. In vivo* and *in vitro* evidence suggests that *P. aeruginosa* produce biofilm within the airways of chronic CF pulmonary infection patients,[2-5] which is a protective barrier around the bacterial cells and limits exposure to oxidative radicals, antibiotics, and phagocytes [6]. Bacterial biofilms play a relevant role in persistent infections, which are rarely eradicated with antimicrobial therapy [7].

Despite the evidence of *P. aeruginosa* grown in the airways of CF patients in biofilm form, the susceptibility profile of the bacterium is usually evaluated, *in vitro,* in the planktonic state. However, the planktonic susceptibility profile may not represent the actual susceptibility of the bacteria [7]. To overcome the potential shortfalls of traditional (planktonic) microbiological methods to evaluate susceptibility, biofilm models have been proposed to access susceptibility of *P. aeruginosa in vitro*[8].

Macrolide antibiotics are being evaluated for the treatment of chronic lung inflammatory diseases, including diffuse panbronchiolitis, CF, chronic obstructive pulmonary disease, and asthma. Although macrolides have no antimicrobial activity against *P. aeruginosa* at therapeutic concentrations, there is great interest in the evaluation of treatments of CF patients with these antibiotics, at least as complementary therapy [9-11]. Anti-inflammatory activity of macrolides has been showed in many studies, including clinical trials [12-17]. Macrolides have also proved to present potential effects on inhibition of bacterial biofilm with reduction of bacterial virulence factor when used in sub-inhibitory concentrations [18]. In the present study, we evaluated the *in vitro* action of macrolides in combination with anti-pseudomonal agents on biofilm-grown *P. aeruginosa* recovered from CF patients.

## Results

The MIC_50_ and MIC_90_ (mg/L) for the 64 isolates were as follows: ceftazidime (CAZ) 2 and 16; ciprofloxacin (CIP) 0.5 and 16; tobramycin (TOB) 2 and 64; imipenem (IPM) 1 and 16; meropenem (MEM) 0.5 and 4; respectively. BIC_50_ and BIC_90_ (mg/L) for all isolates were as follows: CAZ 8 and 256; CIP 1 and 64; TOB 4 and 64; IPM 16 and 256; MEM 2 and 32, respectively. There was a statistical significant difference between MIC and BIC values of isolates for all antibiotics tested (Table 1).

**Table 1:** Anti-pseudomonal agents *in vitro *activity against *P. aeruginosa *(n = 64) in planktonic and in biofilm conditions

**Antimicrobial Agent**	**Range MIC/ BIC**	No. of isolates inhibited by different MIC/BIC values (mg/L) (n=64)										**MIC**_**50**_**/ BIC**_**50**_**(mg/L)**	**MIC**_**90**_**/ BIC**_**90**_**(mg/L)**	***P*value**
		**≤0.5**	**1**	**2**	**4**	**8**	**16**	**32**	**64**	**128**	**≥256**			
**CAZ**	0.5-256/ 0.5-256	3/5	16/10	22/11	8/1	6/3	3/6	2/4	3/4	0/4	1/12	2/8	16/256	<0.001
**CIP**	0.5-128/ 0.5-256	42/31	3/10	7/4	2/2	1/3	5/3	3/4	0/4	1/1	0/2	0.5/1	16/64	0.016
**TOB**	0.5-256/ 0.5-256	9/4	17/6	18/13	7/11	1/7	1/10	1/4	3/4	0/1	7/4	2/4	64/64	0.008
**IPM**	0.5-128/ 0.5-256	21/8	17/1	6/2	5/9	7/6	6/11	1/6	0/5	1/5	0/11	1/16	16/256	<0.001
**MEM**	0.5-64/ 0.5-256	38/21	7/0	7/18	6/7	4/10	0/0	1/2	1/0	0/1	0/5	0.5/2	4/32	<0.001

Detailed legend: CAZ - ceftazidime, CIP - ciprofloxacin, TOB - tobramycin, IPM - imipenem, MEM - meropenem, *P* – statistical significance (< 0.05), MIC – minimal inhibitory concentration, BIC – biofilm inhibitory concentration.

The number of “non-susceptible” (“Resistant” - “R” - plus “Intermediate” - “I”) isolates according to MIC and BIC for each antibiotic was as follows: CAZ 9/64 (14.1%) and 24/64 (37.5%); CIP 19/64 (29.7%) and 23/64 (36%); TOB 13/64 (20.4%) and 30/64 (46.8%); IPM 15/64 (23.4%) and 44/64 (68.8%); MEM 6/64 (9.4%) and 18/64 (28.1%), respectively. There was a statistical significant difference between the susceptibility category of isolates for all antibiotics tested, except for CIP (CAZ: *P* = 0.001, CIP: *P* = 0.234, TOB*: P* = 0.001, IPM: *P* < 0.001, MEM: *P* = 0.005).

The macrolide MIC values were tested for all isolates. Both azithromycin (AZM) (range 32 - 4096) and clarithromycin (CLR) (range 128 - 4096) presented a median MIC of 512 mg/L. MIC_50_ and MIC_90_ (mg/L) for all isolates were 512 and 1024 for AZM; 512 and 4096 for CLR, respectively.

**Figure 1:**
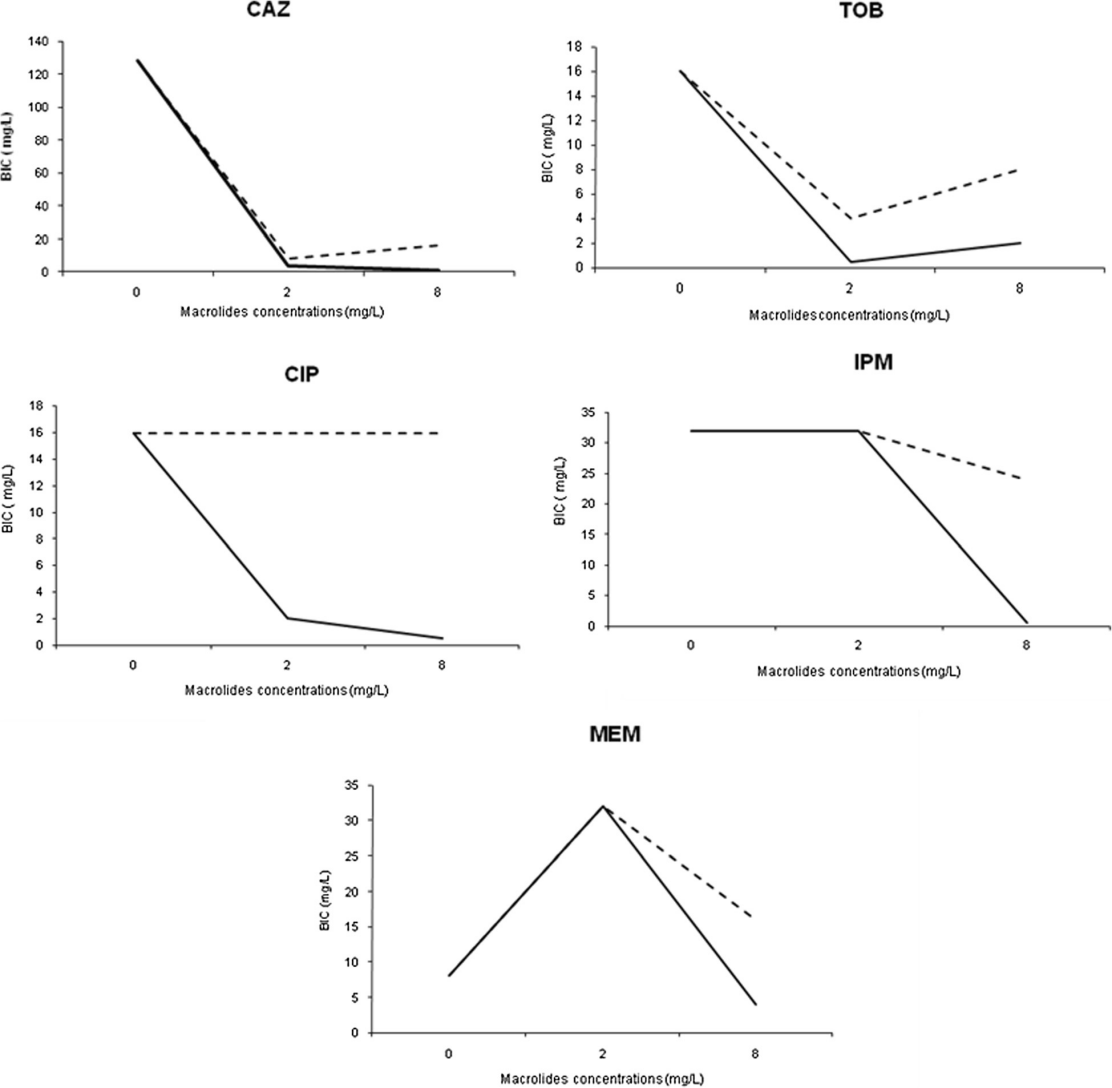
**Azithromycin and clarithromycin action on biofilm inhibitory concentration (BIC) of non-susceptible *P. aeruginosa *isolates combined with anti-pseudomonal agents.** Detailed legend: CAZ - ceftazidime, CIP - ciprofloxacin, TOB - tobramycin, IPM - imipenem, MEM - meropenem, CLR - clarithromycin, AZM – azithromycin. Results are expressed as median of BIC. Solid lines represent association with AZM; dashed lines represent association with CLR.

The non-suscetible isolates according to BIC results were included in the macrolide combination assay (MCA) with CAZ (28 isolates – median BIC 128 mg/L), CIP (23 isolates – median BIC 16 mg/L), TOB (30 isolates – median BIC 16 mg/L), IPM (44 isolates – median BIC 32 mg/L), and MEM (18 isolates – median BIC 8 mg/L). When 2 mg/L of CLR was associated with the anti-pseudomonal agents, the median BIC values were significantly reduced for CAZ (*P* < 0.001) and TOB (*P* < 0.001), but not for CIP (*P* =1.000), IPM (*P* =1.000), and MEM (*P* = 1.000). At higher CLR concentration (8 mg/L), BIC values significantly reduced when associated with CAZ (*P* < 0.001), but not when associated with CIP (*P =* 1.000), TOB (*P =* 0.108), IPM (*P* = 1.000), and MEM (*P =* 1.000). In the presence of 2 mg/L of AZM in combination with the anti-pseudomonal agents, the median BIC values were reduced significantly for CAZ (*P* = 0.001), CIP (*P =* 0.009), and TOB (*P =* 0.001), but not when associated with IPM (*P* = 1.000) and MEM (*P* = 1.000), while the presence of 8 mg/L of AZM in association with all antibiotics showed reduction in median BIC values for all antibiotics tested (CAZ: *P* < 0.001, CIP: *P* < 0.001, TOB: *P < *0.001, IPM: *P <*0.001, MEM: *P <*0.001) (Figure 1).

**Figure 2:**
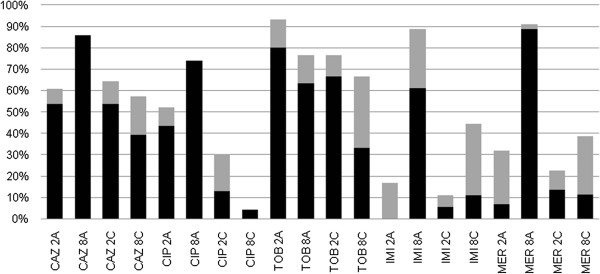
**Inhibitory Quotient (IQ) of combinations of macrolide antibiotics to anti-pseudomonal agents against *P. aeruginosa *isolates.** Detailed legend: CAZ 2AZM – ceftazidime with 2 mg/L of azithromycin, CAZ 8AZM - ceftazidime with 8 mg/L of azithromycin, CAZ 2CLR - ceftazidime with 2 mg/L of clarithromycin, CAZ 8CLR - ceftazidime with 8 mg/L of clarithromycin, CIP 2AZM – ciprofloxacin with 2 mg/L of azithromycin, CIP 8AZM - ciprofloxacin with 8 mg/L of azithromycin, CIP 2CLR - ciprofloxacin with 2 mg/L of clarithromycin, CIP 8CLR - ciprofloxacin with 8 mg/L of clarithromycin, TOB 2AZM – tobramycin with 2 mg/L of azithromycin, TOB 8AZM - tobramycin with 8 mg/L of azithromycin, TOB 2CLR - tobramycin with 2 mg/L of clarithromycin, TOB 8CLR - with 8 mg/L of clarithromycin, IPM 2AZM – imipenem with 2 mg/L of azithromycin, IPM 8AZM - imipenem with 8 mg/L of azithromycin, IPM 2CLR - imipenem with 2 mg/L of clarithromycin, IPM 8CLR - imipenem with 8 mg/L of clarithromycin, MEM 2AZM – meropenem with 2 mg/L of azithromycin, MEM 8AZM – meropenem with 8 mg/L of azithromycin, MEM 2CLR – meropenem with 2 mg/L of clarithromycin, MEM 8CLR – meropenem with 8 mg/L of clarithromycin. STRONG IQ (Black bar) means that there was a reduction in biofilm inhibitory concentration (BIC) when macrolides combination was tested and the isolates changed its profile from “Resistant” to “Susceptible”; WEAK IQ (Grey bar) means that there was a reduction in BIC value when the isolate profile changed from “Resistant” to “Intermediate”.

CLR at 2 mg/L presented strong inhibitory quotient (IQ) when associated with TOB (66.7% of isolates) and CAZ (57.1% of isolates). CLR at 8 mg/L presented strong IQ when associated with CAZ (57.1% of isolates). AZM at 2 mg/L presented a strong IQ when associated with CAZ (50% of isolates), CIP (43.5% of isolates), and TOB (86.7% of isolates). Moreover, 8 mg/L of AZM in combination with all anti-pseudomonal agents tested presented the highest proportion of isolates with strong IQ for all antibiotics tested: CAZ (75%); CIP (73.9%); TOB (70%); IPM (88.6%); and MEM (61.1%) (Figure 2).

A total of 19 (29.7%) isolates presented the mucoid phenotype, but no statistical significant differences in the susceptibility profile of mucoid and non-mucoid isolates were found for the antibiotics tested in the different conditions performed in this study (MIC, BIC and MCA).

The repeatability of the assays demonstrated a coefficient of variation (CV) of MIC and BIC for CAZ, CIP, IPM, MEM, and TOB of 10.21 and 9.45, 7.09 and 8.46, 14.74 and 2.13, 7.70 and 3.94, 10.01 and 8.51, respectively. When macrolides were associated, the highest CV was 20.12% for CAZ with 8 mg/L of CLR and the lowest was 0% for TOB with 2 and 8 mg/L of CLR.

## Discussion

Bacteria in biofilm are more prone to resist treatment with antibiotics and to evade the action of immune system cells. The present study observed a significant difference between MIC in planktonic and in biofilm growth conditions. BIC values were considerably higher than the conventional MIC values for all anti-pseudomonal antibiotics tested in our study as also found by Moskowitz and collaborators [19]. MEM proved to be the most active antibiotic regardless the growth condition, CAZ proved to be the second most active antibiotic in planktonic conditions of growth, whereas CIP was the second most active antibiotic in biofilm conditions. *In vitro* studies have indicated that CIP is one of the most active agents against bacterial biofilm of *S. aureus* and *P. aeruginosa*. This is possibly related to the fluoroquinolones ability to penetrate into biofilms killing non-growing bacteria [20-22]. As expected, all isolates were resistant to AZM and CLR.

The principal finding of our study was that non-susceptible *P. aeruginosa* exposed to macrolides at sub-inhibitory concentrations became susceptible to a variety of anti-pseudomonal agents (CAZ, CIP, IPM, MEM, and TOB) in biofilm conditions. It is of note that in many associations we found a strong IQ between anti-pseudomonal agents and macrolides. The impact of tobramycin/clarithromycin and ceftazidime/clarithromycin co-administration on *P. aeruginosa* biofilms was also observed in studies of Tré-Hardy and collaborators [23,24]. Other study showed that the biofilm was strongly affected by the presence of clarithromycin, and, in its presence, amikacin MIC lower than those obtained in the absence of clarithromycin [25].

In our study, co-administration of AZM at 8 mg/L presented considerable impact when associated with all anti-pseudomonal agents tested (CAZ, CIP, IPM, MEM, and TOB) on *P. aeruginosa* biofilms from CF patients. Although AZM has no bactericidal effect on *P. aeruginosa*, it was shown that AZM retards the formation of biofilms and blocks the bacterial *quorum sensing* involved in the production of biofilms [26-28]. The use of AZM to treat chronic infections of *P. aeruginosa* in the lungs of CF patients has been gaining favour due to the improved outcome of CF patients treated with this antibiotic [29,30].

Synergistic and additive activities were noted when AZM and CLR were paired with conventional antimicrobial agents for *P. aeruginosa* strains in the study of Saiman and collaborators. Overall, combinations were more active against CF isolates than against non-CF isolates and more active against mucoid strains than against non-mucoid strains [31]. However, in our study no significant difference in the macrolides combination assay was observed when we compared mucoid with non-mucoid *P. aeruginosa* clinical isolates.

Interpretative criteria of susceptibility are not standardized for the combination assay in biofilm conditions and this is the main limitation of our study. Therefore, one must be aware that the biofilm susceptibility testing and the macrolide combination assay proposed in our study need further clinical validation for applying it in microbiology laboratories.

## Conclusions

In conclusion, *P. aeruginosa* clinical isolates from CF patients within biofilms are highly resistant to antibiotics and macrolides may be useful as adjunctive therapy as they proved to augment the *in vitro* activity of anti-pseudomonal agents.

## Methods

### Bacterial isolates

A total of 64 *P. aeruginosa* isolates were collected from the sputum of 34 (20 male and 14 female) CF patients attending at the Cystic Fibrosis Centre in Hospital de Clínicas de Porto Alegre, Brazil, from December 2005 to July 2008. The median age of patients was 13 years (range 2 - 30) and the majority of patients presented positive sputum culture for *P. aeruginosa* for at least 5 years. In most children cases, the sputum was obtained only after respiratory physiotherapy. Sputum samples were cultured quantitatively by standard microbiological methods [32]. Isolates of *P. aeruginosa* obtained from the sputum culture were stored at −80°C. *P. aeruginosa* ATCC 27853 was used as quality control for the anti-pseudomonal agents, *S. aureus* ATCC 25923 was used as quality control for the macrolides agents, and PA01 was used as reference of biofilm-forming bacteria.

### Susceptibility tests

#### Antimicrobial agents

Stock solution of antibiotics were prepared following the instructions of the manufacturer (Sigma-Aldrich® Co, St Louis, USA) and stored at −80°C until use. Working solutions were prepared in cation-adjusted Mueller-Hinton broth (CAMHB) (Becton Dickinson, Sparks, MD) at 512 mg/L for CAZ, CIP, TOB, IPM, and MEM. AZM and CLR working solutions were prepared at 8192 mg/L. From these working solutions serial twofold dilutions were prepared in CAMHB and distributed in a 96-well microtiter plate.

#### Minimal inhibitory concentration (MIC) and biofilm inhibitory concentration (BIC)

MIC values were determined by broth microdilution using the twofold dilution method according to the Clinical and Laboratory Standards Institute (CLSI) guidelines [33]. The antibiotic concentrations tested ranged from 0.5 to 256 mg/L for the anti-pseudomonal antibiotics CAZ, CIP, TOB, IPM, and MEM; and from 2 to 4096 mg/L for the macrolides AZM and CLR.

BIC values were determined as previously described [19]. Prior to testing, the organisms were subcultured in trypticase soy broth with 5% KNO_3_ and incubated overnight after retrieval from −80°C. Bacteria were re-subcultured in MacConkey agar (bioMèrieux®, France) and incubated overnight. A bacterial suspension in CAMHB containing 5% KNO_3_ was prepared with an inoculum density equivalent to 0.5 McFarland (Densimat, bioMèrieux®). Afterwards, 100 μL were inoculated into all but the negative control of a flat-bottom 96-well microtiter plate. Plates were covered with lids presenting 96 pegs in which the biofilms could build up, followed by incubation at 37°C for 20 h. Peg lids were rinsed three times with sterile saline to remove non-binding cells, placed onto other 96-well flat-bottom microplates containing a range of antibiotic concentrations and incubated for 18 to 20 h at 37°C. Pegs carrying control biofilms were submerged in antibiotic-free medium. After antibiotic incubation, peg lids were again rinsed three times in sterile saline and incubated in fresh CAMHB in a new microplate and centrifugated at 805 X **g** for 20 min. The peg lid was discarded and replaced by a standard lid. The optical density (OD) at 650 nm was measured on a microtiter plate colorimeter before and after incubation at 37°C for 6 h (OD_650_ at 6 h minus OD_650_ at 0 h). Biofilm formation was defined as a mean OD_650_ difference ≥ 0.05 for the biofilm control. The BIC values were defined as the lowest concentration without growth. CLSI criteria [34] were used to classify the isolates as ¨Susceptible¨ (“S”), ¨Intermediate¨ (“I”) or ¨Resistant¨ (“R”).

#### Macrolide combination assay (MCA) and inhibitory quotient (IQ)

Only isolates with a BIC value in “R” or "I” classification according to CLSI interpretative criteria [34] for CAZ, CIP, TOB, IPM, and MEM were used in the MCA and IQ.

MCA was performed in a 96-well microplate containing CAZ, CIP, TOB, IPM, or MEM in twofold dilutions in addition to macrolides at sub-inhibitory concentrations [35]. With the purpose to assign activity of AZM and CLR in combination with the antibiotics and to better evaluate susceptibility changing category, we established an inhibitory quotient (IQ). IQ is the quotient of the maximum antibiotic serum concentration and the BIC value of each antibiotic in combination with the macrolide. IQ categorization for CAZ, CIP, TOB, IPM, and MEM to evaluate the activity of macrolides in different concentrations against resistant *P. aeruginosa* isolates was as follows: strong IQ (IQ ≥ 2, except for CIP, whose IQ was ≥ 1), weak IQ (IQ = 0.5), or non-inhibition (IQ ≤ 0.5). Strong IQ means that there was a reduction in BIC when macrolide combination was tested and the isolates changed their profile from “R” to “S”; weak IQ means that there was a reduction in the BIC value when the isolate profile changed from “R” to “I”; and non-inhibition means no change in the bacteria antibiotic susceptibility profile [36].

All assays were performed four times. Mean values of the four repetitions, standard deviations, and CV were calculated and the mean value was considered the value which was then used to categorize the isolates as “R”, “I” or “S”.

The susceptibility profile of mucoid and non-mucoid isolates was evaluated under the different conditions performed in this study (MIC, BIC and MCA).

#### Statistical analysis

The Wilcoxon signed ranks test was used for statistical analysis of quantitative values of MIC and BIC. McNemar-Bowker test was used to evaluate the categories of the results obtained (“S”, “I” and “R”) by the standard technique and the technique in biofilm. *P* < 0.05 indicated statistical significance.

#### Ethics aspects

The bacterial isolates were obtained from clinical specimens sent for routine culture in the Microbiology Unit of Hospital de Clínicas de Porto Alegre. The information was compiled in order to respect the privacy of patients; written informed consent for participation in the study was obtained from participants or, where participants were children, from a parent or guardian. This study was approved by the Ethics Committee in Research of Hospital de Clínicas de Porto Alegre (project number 06 - 406).

## Competing interests

The authors have no competing interests to declare.

## Authors' contributions

LL conceived the study design and coordinated the study, carried out the microdilution methods, performed the statistical analysis and drafted the manuscript. DCP carried out the microdilution methods, performed the statistical analysis and drafted the manuscript. RMP participated in the design of the study and drafted the manuscript. APZ analysed and drafted the manuscript. ALB conceived the study design, coordinated the study and drafted the manuscript. All authors read and approved the final manuscript.
